# *Pseudomonas aeruginosa* Adaptation to Lungs of Cystic Fibrosis Patients Leads to Lowered Resistance to Phage and Protist Enemies

**DOI:** 10.1371/journal.pone.0075380

**Published:** 2013-09-19

**Authors:** Ville-Petri Friman, Melanie Ghoul, Søren Molin, Helle Krogh Johansen, Angus Buckling

**Affiliations:** 1 Department of Zoology, University of Oxford, Oxford, United Kingdom; 2 Biosciences, University of Exeter, Penryn, United Kingdom; 3 Department of Systems Biology, Technical University of Denmark, Lyngby, Denmark; 4 Department of Clinical Microbiology,Rigshospitalet, København, Denmark; Leiden University, Netherlands

## Abstract

Pathogenic life styles can lead to highly specialized interactions with host species, potentially resulting in fitness trade-offs in other ecological contexts. Here we studied how adaptation of the environmentally transmitted bacterial pathogen, *Pseudomonas aeruginosa*, to cystic fibrosis (CF) patients affects its survival in the presence of natural phage (14/1, ΦKZ, PNM and PT7) and protist (*Tetrahymena thermophila* and *Acanthamoebae polyphaga*) enemies. We found that most of the bacteria isolated from relatively recently intermittently colonised patients (1–25 months), were innately phage-resistant and highly toxic for protists. In contrast, bacteria isolated from long time chronically infected patients (2–23 years), were less efficient in both resisting phages and killing protists. Moreover, chronic isolates showed reduced killing of wax moth larvae (*Galleria mellonella*) probably due to weaker *in vitro* growth and protease expression. These results suggest that *P. aeruginosa* long-term adaptation to CF-lungs could trade off with its survival in aquatic environmental reservoirs in the presence of microbial enemies, while lowered virulence could reduce pathogen opportunities to infect insect vectors; factors that are both likely to result in poorer environmental transmission. From an applied perspective, phage therapy could be useful against chronic *P. aeruginosa* lung infections that are often characterized by multidrug resistance: chronic isolates were least resistant to phages and their poor growth will likely slow down the emergence of beneficial resistance mutations.

## Introduction

Parasitic lifestyles often demand complex adaptations to circumvent host defences [1]–[3]. As a result, host-parasite coevolutionary arms races are likely to promote specialization in parasitic organisms [3], [4]. Intracellular bacterial pathogens are one extreme case of parasite specialization. For example, the small genomes of *Mycoplasma* and *Rickettsia* are thought to arise as a result of evolutionary transition from free-living environmental bacteria to more permanent, pathogenic associate of its host species [5], [6]. Commonly lost properties include genes related to bacterial energy metabolism and amino acid synthesis: traits that become non-vital for pathogen survival because the host can provide a surplus of nutrients [6]. Moreover, immune evasion may be partly responsible for loss of important virulence genes [7]–[9]. Interestingly, similar loss of genes and functions has also been observed with facultative, environmentally transmitted opportunistic pathogens [8], [10]–[12], which are continuously released from infected hosts to external environment before being picked up by new hosts (e.g., in sputum or faeces of cystic fibrosis (CF) and cholera patients, respectively). While there has been considerable research into the consequences of within-host adaptation for pathogen virulence, [8], [10]–[12], the impact of within-host specialisation on the opportunistic pathogen survival in external environments is less clear.

Many environmentally transmitted opportunistic pathogens are generalists in the sense that many traits confer fitness advantages both inside and outside the host (dual-use traits), and hence adaptation to one environment may indirectly increase fitness in the other [13], [14]. Many studies have shown positive correlation between virulence and survival in environmental reservoirs. For example, resistance against degradative enzymes of amoeba can help bacteria to resist attack by mammalian macrophages [15]–[17], while proteases responsible for damaging host tissues often have nutritional role in external environments [13]. This is, however, not always the case and some pathogens can experience conflicting selection pressures between external and internal environments [18]–[20], where adaptation to one environment leads to reduced fitness in the other [21], [22]. As a result, adaptation to within-host environments could reduce pathogen survival in external environments through trade-offs or vice versa.

Here we investigated these hypotheses by comparing the survival and resistance of 28 *P. aeruginosa* strains isolated from the lungs of CF patients (Table 1) to different natural protist (*Tetrahymena thermophila* and *Acanthamoebae polyphaga,* Table 2) and phage enemies (14/1, ΦKZ, PNM and PT7, Table 2). *P. aeruginosa* is a good example of an environmentally transmitted, opportunistic bacterial pathogen with broad host spectrum [23] and currently about 10% of hospital-acquired infections in the European Union hospitals are caused by *P. aeruginosa* [11], where the bacterium often establishes itself in already compromised patients, such as those with CF or hospitalized in intensive care units [11], [12]. Recent studies involving whole-genome analysis have demonstrated that *P. aeruginosa* adaptation to human lung environment leads to drastic changes including reduced growth, lowered motility, down regulation of sigma factors, increased biofilm formation, loss of quorum-sensing [8], [10], [12]. While most of these factors are important for bacterial anti-predatory defences [15], [24], they can also affect the *P. aeruginosa* virulence [8], [10], [12], [25], [26].

**Table 1:** Bacterial strains used in this study.

Strain No.	Patient No.	Source	Year obtained	Infection type	Duration of infection	Colony type
1	1	Copenhagen CF Center Rigshospitalet	2004	Intermittent colonisation	6 months	Yellow
2	2	Copenhagen CF Center Rigshospitalet	2005	Intermittent colonisation	6 months	Yellow
3	2	Copenhagen CF Center Rigshospitalet	2006	Intermittent colonisation	12 months	Yellow
4	2	Copenhagen CF Center Rigshospitalet	2006	Intermittent colonisation	25 months	Yellow
5	3	Copenhagen CF Center Rigshospitalet	2005	Intermittent colonisation	16 months	Yellow
6	3	Copenhagen CF Center Rigshospitalet	2006	Intermittent colonisation	22 months	Yellow
7	4	Copenhagen CF Center Rigshospitalet	2005	Intermittent colonisation	1 month	Yellow
8	5	Copenhagen CF Center Rigshospitalet	2004	Intermittent colonisation	4 months	Yellow
9	6	Copenhagen CF Center Rigshospitalet	2007	Intermittent colonisation	4 months	White
10	7	Copenhagen CF Center Rigshospitalet	2006	Intermittent colonisation	< 1 month	Yellow
11	8	Copenhagen CF Center Rigshospitalet	1978	Chronic infection	2 years	Yellow
12	8	Copenhagen CF Center Rigshospitalet	1985	Chronic infection	9 years	Yellow
13	8	Copenhagen CF Center Rigshospitalet	1987	Chronic infection	11 years	Yellow
14	8	Copenhagen CF Center Rigshospitalet	1991	Chronic infection	15 years	White
15	9	Copenhagen CF Center Rigshospitalet	1984	Chronic infection	1 year	Yellow
16	9	Copenhagen CF Center Rigshospitalet	1988	Chronic infection	5 years	White
17	9	Copenhagen CF Center Rigshospitalet	2004	Chronic infection	21 years	White
18	10	Copenhagen CF Center Rigshospitalet	1997	Chronic infection	4 years	White
19	10	Copenhagen CF Center Rigshospitalet	1999	Chronic infection	6 years	Yellow
20	10	Copenhagen CF Center Rigshospitalet	2004	Chronic infection	11 years	White
21	11	Copenhagen CF Center Rigshospitalet	1979	Chronic infection	3 years	Yellow
22	11	Copenhagen CF Center Rigshospitalet	1984	Chronic infection	8 years	Yellow
23	11	Copenhagen CF Center Rigshospitalet	1985	Chronic infection	9 years	Yellow
24	11	Copenhagen CF Center Rigshospitalet	1994	Chronic infection	18 years	White
25	11	Copenhagen CF Center Rigshospitalet	1999	Chronic infection	23 years	White
26	12	Copenhagen CF Center Rigshospitalet	1991	Chronic infection	1 year	White
27	13	Copenhagen CF Center Rigshospitalet	1986	Chronic infection	6 years	White
28	13	Copenhagen CF Center Rigshospitalet	2002	Chronic infection	22 years	White

**Table 2:** Protist and phage species used in this study.

Strain	Enemy type	Source	Family
Tetrahymena thermophila, CCAP 1630/1U	Ciliate protist	Unknown, Claff (1939)	Tetrahymenidae
Acanthamoeba polyphaga, CCAP 1501/18	Amoeba protist	Unknown, Rowbotham (1985)	Amoebadie
14/1	Phage	Sewage water, Regensburg, Germany, 2000	Myoviridae A1
ΦKZ	Phage	Sewage water, Kazakhstan, 1975	Myoviridae A1
PNM	Phage	Mtkvari River, Tbilisi, Georgia, 1999	Podoviridae C1
PT7	Phage	Lake Ku, Tibilisi, Georgia, 1999	Myoviridae A1

Bacteria were categorised in two classes according to the duration of infection: isolates from relatively short-term, intermittent colonisations (1–25 months), and isolates from much older chronic infection (2–23 years). This classification is based on the following differences: strains from intermittent colonisations can be eradicated with antibiotics and no antibodies are yet found from patient serum, while in chronic infections, pathogens are discovered continuously from sputum samples for >6 months and/or there is a significant antibody response against *P. aeruginosa* [27]. We used isolates from 7 intermittently colonised and 6 chronically infected patients. When patients had been sampled repeatedly at different time points, patient mean values were used. Differences in bacterial resistance against natural enemies were measured in short-term growth assays in the absence and presence of all different enemy species. Bacterial virulence was measured *in vivo* in wax moth host, and a few potential virulence traits (motility, biofilm formation, protease expression) were quantified with standard microbiological assays.

Our results show that bacteria isolated from chronic infections are more susceptible to both phage and protist predation and less virulent in wax moth compared to bacteria isolated from intermittent colonisations. These results complement a recent study reporting less resistance to phages of clinical isolates compared with environmental isolates of *P. aeruginosa* [28] and suggest that within host adaptation could lead to poorer transmission if bacteria released to external environments are exposed to natural microbial enemies before infecting new hosts. From a more applied perspective, phage therapy - the use of viruses to specifically kill only the disease-causing bacteria - could be particularly effective against more phage susceptible bacteria from chronic *P. aeruginosa* lung infections that cannot anymore be eradicated with antibiotics.

## Materials and Methods

### Ethics statement

The present project is in compliance with the Helsinki Declaration (Ethical Principles for Medical Research Involving Human Subjects). Strains were collected from sputum as part of the patients’ routine care, without any additional sampling. The ethic committee in ‘‘RegionH The Capital Region of Denmark’’ was consulted, specifically approved this study and declared that patient informed consent was not needed.

### Study species and culture conditions

A total of 28 different *P. aeruginosa* isolates, two different protist strains (*T. thermophila* and *A. polyphaga*) and 4 different phage species (14/1, ΦKZ, PNM and PT7) were used in the experiments (strains listed in Tables 1 and 2). All phage and protist species used in this study are commonly found in natural environments [29]–[31], where they likely cause high bacterial mortality and hence strong selection for resistance [28], [32]. Bacterial strains were obtained from Copenhagen CF Center Rigshospitalet (partly described earlier [33]) and divided into two categories on the basis of following guidelines [27]: bacterial strains isolated from relatively young, intermittent colonisations, that can be eradicated with antibiotics while no antibodies are yet found from patient serum, and bacteria isolated from chronic infections, where pathogens are continuously discovered from sputum samples for >6 months and/or there is a significant antibody response against *P. aeruginosa* [27].

Bacterial stocks were prepared by growing bacteria overnight on KB agar plates, after bacterial mass was streaked and mixed to M9 salt solution with sterile loops (VWR). All clones were diluted to the same densities (approximately 0.2 optical density at 600 nm wavelength, equalling approximately 9 × 10^7^±150 cells mL^−1^). Colony colour and morphology of every strain was recorded by plating bacteria on King’s B (KB) medium agar plates (M9 salt solution supplemented with 10 g L^−1^ glycerol, 20 g L^−1^ proteose peptone and 12 g L^−1^ agar). Both protist stocks were cultured with non-living resource prior the experiments; Peptone Yeast medium (PPY; 20 g L^−1^ Peptone and 2.5 g L^−1^ of Yeast extract) was used for *T. thermophila* and Proteose Peptone Glucose medium (PPG; Page’s Amoeba Saline solution supplemented with 15 g L^−1^ Peptone and 18 g L^−1^ of D-glucose) for *A. polyphaga*. Both protist stock solutions were diluted ten-fold with M9 buffer to obtain inoculums for growth measurements. Phage stock solutions were prepared by growing all frozen phages (–80°C) with PAO1 (ATCC #15692) strain in liquid KB medium for 24h before chloroforming (10% volume) and centrifugation to purify phages from bacteria. Phages were stored at 4°C. Phage densities were estimated by diluting and plating three independent stock solutions of each phage species onto lawns of PAO1 bacterial strain. The mean ±s.e. density (PFU, i.e., plaque forming units) for each phage species was as follows: 14/1: 591,666,666±17638342; ΦKZ: 305,000,000±94,648,472; PNM: 205,000,000 ±71,821,538; PT7: 1,065,000,000±150,582,203. All species were always grown at 28°C.

### Bacterial growth measurements in the absence and presence of phage and protist enemies

The growth of all bacterial strains (starting densities of approximately 10^5^ cells mL^−1^; see methodology for equalizing inoculum above) was measured both in the absence and presence of every single enemy in 200 µL of fresh 10% KB medium by using a photo spectrometer (Bio-Tek Instruments, Inc.,Winooski, VT, USA, OD 600 nm). In the enemy-cocultures, small inoculums of every enemy were subsequently added to microplate wells immediately after inoculating bacteria: 20–25 cells of *A. polyphaga*, 10–15 cells of *T. thermophila*, 591666 particles of 14/1 phage, 305000 particles of ΦKZ phage, 205000 particles of PNM phage and 1065000 particles of PT7 phage. All growth measurements were replicated twice and run for 91 hours.

### Bacterial life-history trait measurements

We determined the resistance to phages by streaking independent bacterial clones across a line of each phage (40 μL) that had previously streaked and dried on a KB agar plate. A colony was scored as resistant if there was no inhibition of growth by the phage [34].

Bacterial biofilm was measured by growing all clones for 48 h in 10% KB, after, 50 µl of 1% crystal violet solution was added to the microplate wells and rinsed off with distilled water after 10 minutes. The remaining crystal-violet attached to bacteria was dissolved in 96% ethanol and the amount of biofilm formed measured as OD at 600 nm [35].

Motility was assessed by sticking a trace inoculum (∼2 µL) of each clone with a sterile loop (VWR) to the centre of a semi-solid agar plate (as described above, except 0.7% agar). The plates were photographed after 48h and the colonised area was determined with ImagePro Plus 4.5 software (Media Cybernetics).

Protease expression was measured as a zone of hydrolysis on KB agar plates incremented with 20 g L^−1^ of skim milk powder (Merck). Bacteria were first grown in 10% KB for 48 h, before inserting 10 µL of solution on sterilised filter disk (∅ 6 mm, Whatman). Protease expression was determined as a zone of hydrolysis after 24h incubation.

Bacterial virulence was measured in wax moth larvae (*Galleria mellonella,* Lepidoptera; Pyralidae; Livefood UK). Bacterial virulence measured in *G. mellonella*, mammals and mammalian cell cultures have been found to correlate positively, making it an ideal model host for general virulence testing [36], [37]. All larvae were weighed before infection to achieve equal mean weights between treatments (bacterial group: *F*_4, 331_ = 1.5, *P* = 0.19). Eight larvae per clone were injected with 30 µL (on average 3×10^5^±150 CFU) of bacterial solution between the abdominal segments 6 and 7 with a 1 mL Terumo syringe. Sixteen larvae were injected with 30 µL of M9 buffer to control the damage caused by the injection itself. After infection, larvae were placed on individual wells of 24-well cell culture plates and survival monitored at 2-hour intervals for 3 days at 28°C. Larvae were defined as dead when they did not respond to touch with forceps. Larvae that were still alive after 5 days from infection were given time-to-death value of 141 hours.

### Statistical analyses

A General linear mixed model (GLMM) was used to analyse differences in bacterial growth in the absence and presence of protists and phage enemies. Because some patients had been sampled at repeatedly different time points, patient mean values were used. We first compared the means of patients suffering from intermittent colonisation or chronic infection. Second, we analysed the effect of infection duration by comparing chronic patients with infection lasting either under or over ten years. Lastly, we analysed the significance of bacterial colony type (yellow or white) of chronic patients that had both colony types present (patients no 8–11; Table 1). In these models, bacterial growth was explained with infection type (intermittent vs. chronic), enemy type (no enemy, phage, amoeba, ciliate), time, and additionally, with the length of chronic infection (<10 and >10 years) or colony type (yellow or white). When needed, additional GLMMs were carried out to compare the effect of phage species. In all growth data analyses, populations were used as a subject variables, time as a repeated factor (5 levels; 16, 24, 48, 72 and 91 hours) and patient identity included in the models as a random factor (nested infection type).

Phage resistance was analysed with ANOVA where arcsin transformed patient mean resistances were explained by infection type and phage species. Bacterial motility, biofilm formation, protease expression and virulence were analysed with independent-samples T-tests. Non-parametric Mann-Whitney test was used to analyse bacterial ability to kill protists.

## Results

### Bacterial defence against different enemies

**Figure 1:**
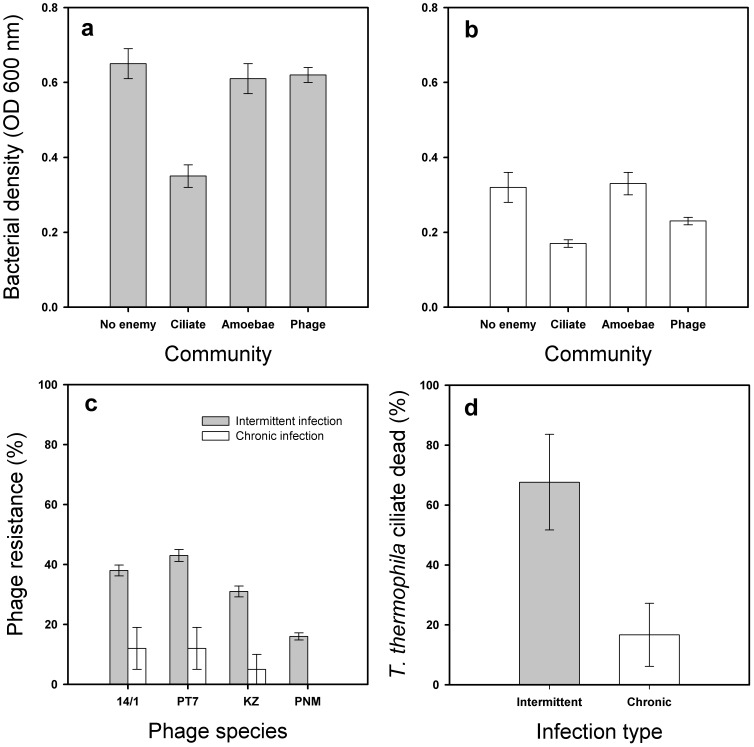
Bacterial growth in the absence and presence of different enemies. The growth of bacteria isolated from patients with intermittent colonisation (a) or chronic (b) CF-lung infection; panels show mean values averaged over time (91 hours). Panels (c) and (d) show bacterial phage resistance (c) and ability to kill ciliate protist (d) for patients suffering from intermittent colonisation or chronic CF-lung infection. Error bars denote ± 1 SEM.

Bacterial growth was affected by the infection type (*F*_1, 58.2_ = 63.8, *P*<0.001) and the presence and type of enemy (*F*_3, 87.7_ = 17.6, *P*<0.001, Figure 1a-b): chronic bacterial isolates grew generally slower (*P*<0.001), and while the ciliate *T. thermophila* clearly reduced bacterial densities regardless of the infection type (*P*<0.001), *A. polyphaga* amoeba and phages had no effect on bacterial densities (*P* = 0.84 and *P* = 0.099, respectively). There was a significant interaction between enemy type and infection type: phages reduced the densities of bacteria isolated from chronic infection regardless of the phage species (infection type × enemy type: *F*_3, 87.7_ = 7.7, *P*<0.001; pairwise comparison: *P* = 0.04; phage species: *F*_3, 15.6_ = 0.37, *P* = 77, Figure 1a-b), while phages had no effect on bacteria associated with intermittent colonisation (pairwise comparison: *P* = 0.59). To further explore this, we next compared chronic patients that had been sampled repeatedly in time, and asked if bacterial growth in the absence and presence of enemies differ between samples isolated from less than ten year long and over ten year long chronic infections. We found similar main effect for the enemy type as above (*F*_3, 52.4_ = 1557, *P*<0.001): amoebae had no effect on bacterial densities (*P* = 0.81), while both ciliates and phages decreased bacterial densities (*P*<0.001 and *P*<0.001, respectively; data not shown). However, the effect of time on bacterial growth was non-significant (*F*_1, 13.7_ = 0.42, *P* = 0.52) regardless of the enemy community type (infection length × enemy type: *F*_3, 52.4_ = 0.21, *P* = 0.889; data not shown), suggesting that the duration of chronic infection does not significantly alter bacterial resistance against ciliate and phage enemies.

Interestingly, we found that 90% of intermittent bacterial isolates formed yellow colonies similar to *P. aeruginosa* PAO1 strain, while chronic isolates formed yellow colonies in 44% and white colonies in 56% of cases (Fisher exact test colony type × infection class, *P* = 0.041, Table 1). As a result, we compared if white and yellow chronic colony types differ in their growth under different enemies. The enemy communities had similar effects as above (*F*_3, 50.3_ = 4.7, *P* = 0.006): amoebae had no effect on bacterial densities (*P* = 0.87), while both ciliate and phages decreased bacterial densities (*P* = 0.005 and *P* = 0.016, respectively). Moreover, white colonies were poorer at growing (*F*_1, 66.9_ = 8.3, *P* = 0.005), and this difference was also dependent on enemy community type (colony type × enemy type: *F*_3, 66.2_ = 2.9, *P* = 0.04): both ciliates and phages were better at reducing the densities of white bacterial colony types (*F*_3, 33.2_ = 4.3, *P* = 0.01; Figure S1c-d) compared to yellow colony types (*F*_3, 42.8_ = 2.6, *P* = 0.06), which suggest that the reduced defence to ciliate and phage enemies was especially attributed to chronic white colony types which were often observed in the later stages of chronic infections (Table 1).

The lack of phage effect on the growth of bacteria isolated from intermittent colonisations can be in part explained by innate phage resistance: bacterial resistance to phages was generally higher with intermittent compared to chronic isolates (*F*_1, 40_ = 6.2, *P* = 0.017; Figure 1c) with different phage species having similar effects (*F*_3, 40_ = 0.24, *P* = 0.86). While the duration of infection did not clearly affect the degree of phage resistance with chronic isolates (*F*_1, 36_ = 3.17, *P* = 0.083; data not shown), the chronic white colony types were less phage resistant compared to yellow colony types (*F*_1, 16_ = 4.3, *P* = 0.05; mean resistance and ± 1 standard error for intermittent and chronic patients respectively: 0.321±0.094 and 0±0.079). This suggests that the lowered phage resistance of chronic isolates was due to lower resistance of white colony types associated with later stages of chronic infections.

The lack of *A. polyphaga* effect on bacterial densities was likely due to *P. aeruginosa* toxicity: all bacterial clones were able to kill *A. polyphaga* amoeba (no living cells observed after 91 h cocultivation; data not shown). In the case of *T. thermophila*, chronic isolates were less toxic compared to intermittent CF-lung isolates (Mann-Whitney *U* = 6, *P* = 0.035, Figure 1d; while no difference in toxicity was found between <10 years and >10 years old isolates (Mann-Whitney *U* = 13, *P* = 0.42; data not shown), or yellow and white colony types (Mann-Whitney *U* = 11, *P* = 0.5; data not shown).

In summary, these results suggest that *P. aeruginosa* clones isolated from chronic CF-lungs grow slower and are more susceptible to phages and ciliates compared to bacteria isolated from intermittently colonised CF-lungs.

### Bacterial virulence trait measurements

**Figure 2:**
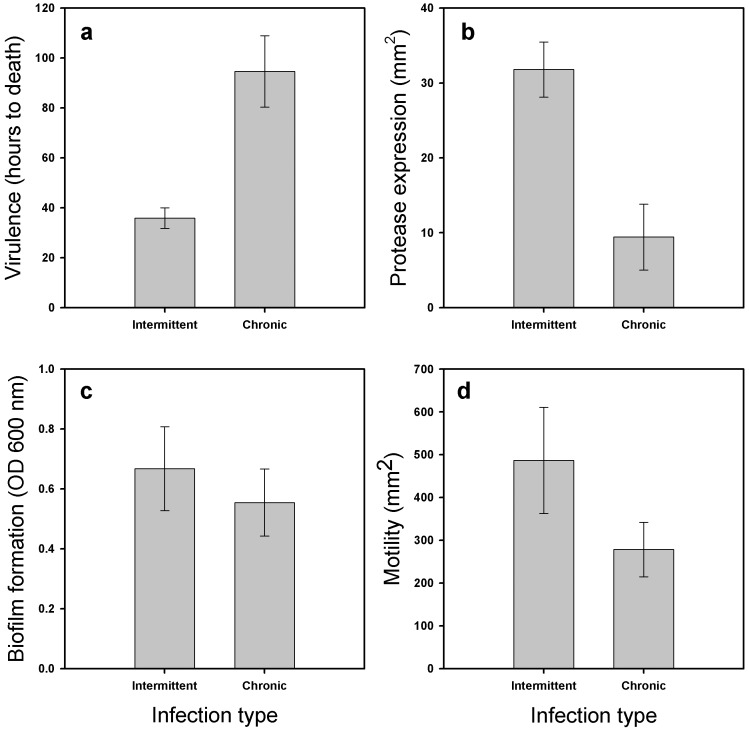
Bacterial trait measurements. Bacterial virulence measured *in vivo* for patients with intermittent colonisation or chronic CF-lung infection (a): virulence is defined as less hours to larval death, more virulent the bacterial strain. Panel (b) shows difference in bacterial protease expression, panel (c) difference in biofilm formation and panel (d) difference in motility for patients suffering from intermittent colonisation or chronic CF-lung infection. Error bars denote ± 1 SEM.

Adaptation to the lung environment decreased bacterial virulence, i.e., there was increased larval survival associated with chronic infection versus intermittent colonisation (infection type: *P* = 0.001, t = –4.5, df = 10; Figure 2a). However, no difference was found between <10 years and >10 years old chronic isolates (*P* = 0.3, t = –1.0, df = 9), or between white and yellow chronic isolates (*P* = 0.26, t = –1.2, df = 6; data not shown). Also, no difference in motility or biofilm formation was found between intermittent and chronic isolates (*P* = 0.21, t = 1.3, df = 10 and *P* = 0.56, t = 0.58, df = 10, respectively, Figure 2b-c), <10 years and >10 years old chronic isolates (*P* = 0.33, t = –1, df = 9 and *P* = 0.92, t = –0.9, df = 9, respectively), or white and yellow chronic isolates (*P* = 0.4, t = –0.89, df = 6 and *P* = 0.83, t = 0.22, df = 6, respectively; data not shown). However, bacterial protease activity was clearly higher with more virulent, intermittent isolates (*P* = 0.003, t = 3.9, df = 10; Figure 2b), while <10 years and >10 years old chronic isolates (*P* = 0.97, t = 0.03, df = 9), and white and yellow chronic isolates (*P* = 0.39, t = –0.9, df = 6) had equally high protease expression (data not shown). These results show that within-host specialisation leads to lowered virulence, which could be mechanistically explained by lowered protease expression.

## Discussion

Here we studied how *P. aeruginosa* adaptation to the lungs of CF patients affects its fitness in external environments. We found that *P. aeruginosa* bacteria isolated from chronic CF-lung infections were less efficient at killing protists and had lower resistance against phages compared to bacteria isolated from intermittent CF-lung colonisations. This difference was partly explained by the high frequency of small, smooth colony variants in chronic infections, which showed poor growth in liquid medium and were especially susceptible to phages. The chronic isolates’ increased susceptibility to ciliate and phage enemies also correlated with lowered virulence in the insect host. However, the duration of chronic infection (<10 vs. >10 years) had minor effects on bacterial fitness, suggesting that prolonged selection within host did not further shape bacterial virulence or resistance to natural enemies. Together these results suggest that *P. aeruginosa* transition from intermittent colonisation to chronic CF-lung infection leads to fitness costs in external environment potentially leading to poorer environmental transmission.

That long-term chronic infection in CF-lung environment was associated with greatly reduced resistance to phages was somewhat surprising, given that adaption to the CF-lung is associated with loss and alteration of range of potential phage receptors [8], [38], which could potentially lead to higher phage resistance. We suggest three possible reasons for our findings. First, phage-imposed selection might be stronger during intermittent colonisations resulting in relatively higher phage resistance. While it has been reported that phages are found in CF-lungs [39], [40], these studies are often based on purely on detection of phage, e.g. by electron microscopy [39] or sequencing [40], and tell little about the densities and biological activity of bacteriophages in CF-lungs. For example, chronic CF-lungs could be dominated by lysogenic prophages that reside most of the time within bacterial genomes and only occasionally induce lytic infection of bacterial cells [41]; in this case, phages might have a relatively small role in regulating bacterial densities despite their prevalence. Second, resistance mutations are often associated with costs [42], and these costs could be increased if chronic infections experience more stressful environments than intermittent colonisations, even if phage-imposed selection is comparable [43]. Finally, selection for phage resistance may be reduced, or actively selected against, in both intermittent colonisation and chronic infections, but loss of resistance (through selection or even drift, if phage resistance is selectively neutral) has occurred to a greater extent in he chronic infections. Consistent with this view, a recent study reported that clinical *P. aeruginosa* isolates have generally lower phage resistance compared to environmental *P. aeruginosa* isolates [28]; In other words, it is possible that phages select for high resistance in environmental reservoirs [44], while the resistance is lost during chronic bacterial infections in the absence of phage selection.

Bacteria isolated from chronic infections were generally less effective at killing *T. thermophila* ciliates (although, surprisingly, ciliates reduced density of both types of infections to the same extent), while both intermittent and chronic isolates were deadly for *A. amoebae*. The chronic CF isolates’ reduced ability to kill ciliates could be explained by reduced production of toxic exo-factors. It has been shown previously that adaptation to CF-lungs reduces rhamnolipid and elastase expression [45], while quorum-sensing deficient mutants typically observed in CF-lungs are less toxic for nematodes [26], *Rhynchomonas nasuta* protists [46] and *T. pyriformis* (Friman *et al.* [In press]). In support, we found that the protease expression of chronic isolates was clearly reduced compared to bacteria isolated from intermittent colonisations. As for phage resistance, protease expression (and toxicity to protists in general) could be lost during chronic infections simply because it is costly having negative effect on bacterial growth [47], because it provokes stronger immune responses towards the pathogen [48], or is selectively neutral and is lost through drift. In contrast to *T. thermophila, A. polyphaga* amoebae were killed in all bacterial co-cultures regardless of the type of infection probably because they were more sensitive to bacterial toxins or because bacteria were simply able to overgrow them in relatively rich nutrient medium.

The rise of small and white *P. aeruginosa* colony types is often connected to enhanced persistence and survival during long-term cystic fibrosis infections [8], [49]. We found that both phages and ciliates were more efficient in reducing the densities of white colony type. Which made this colony type especially vulnerable? One explanation for lowered resistance to ciliates could lie in reduced growth and toxicity. Furthermore, even though yellow and white colony types did not differ in in mean motility or biofilm formation, it is still possible that for example the biofilm structure or the number of pili differed between these colony types. While these both traits has been connected to changes in both *P. aeruginosa* phage resistance and colony morphology [50], further tests are§§ needed to confirm this.

Consistent with previous work [12], adaptation to CF-lung environment also led to reduction in bacterial virulence in wax moth hosts. The loss of virulence was connected to chronic isolates that were poor at growing and expressed weakly proteases, while no clear changes in biofilm formation or motility was observed (lack of effect on motility probably due to pooling of different aged bacterial isolates to patient means for analysis). Whether slow growth and reduced virulence is itself adaptive in terms of helping bacterial cells to go unnoticed by host immune system [51] or resist antimicrobial chemotherapy [52], or if it is a pleiotropic cost associated with other within-host adaptation is unclear.

The observed loss of phage resistance is encouraging in relation to phage therapy: the use of viruses to specifically kill only the disease-causing bacteria [53]–[55]. First, bacterial isolates from chronic infections were especially sensitive to phages. Second, chronic bacterial isolates had poor growth and small population sizes, which will make the emergence of beneficial resistance mutations less likely. Third, if adaptive changes behind within-host adaptation are due to loss of genetic material [56], bacteria might not be able to reverse evolution without horizontal gene transfer. As a result, phage therapy could prove especially effective against chronic bacterial infections that cannot anymore be eradicated with antibiotics. Even though potential complications might rise from the spatial distribution and within- and between-species diversity of bacterial infections [57]–[59], identifying similar fitness trade-offs for bacterial survival between natural and clinical environments could serve as a starting point for developing potential alternatives for antibiotics. For example, within-host specialization could make bacteria more susceptible to bacterium-specific toxins (bacteriocins) that are used in intraspecific bacterial competition in natural environments [47], [60], but might not be needed in the lungs of CF patients.

To conclude, our study shows that environmentally transmitted pathogens’ long-term adaptation to a within-host environment is associated with reduced defence against natural phage and protist enemies. This result supports the general idea that more permanent association with host organisms leads to loss of pathogen virulence, which might play a key role in explaining evolutionary transitions from antagonistic interactions towards commensalisms and mutualism. Moreover, even though the effect of protist predation on bacterial virulence evolution has been studied quite extensively [14]–[18], [20], the consequences of within-host adaptation for opportunistic pathogens’ survival, transmission and prevalence in environmental reservoirs are still poorly understood. For example, from the epidemiological point of view, chronically infected patients could be seen as pathogen “sinks” because their further spread and transmission through environment is limited by their reduced survival in the presence of natural microbial enemies. In the future more effort should thus be put on trying to understand the connectedness and selection in clinical and environmental compartments and how they jointly define the evolution of pathogen virulence [19].

## Supporting Information

Figure S1 **Bacterial growth in the absence and presence of different enemies.** The growth of bacteria isolated from patients with intermittent colonisation (a) or chronic (b) CF-lung infection. Panels (c) and (d) show bacterial growth in the absence and presence of different enemies for yellow (c) and white (d) colony type means for chronic CF-patients. Error bars denote ± 1 SEM.
(TIF)
