# Supplementary figures and images for: Pseudomonas aeruginosa Adaptation to Lungs of Cystic Fibrosis Patients Leads to Lowered Resistance to Phage and Protist Enemies
Source: PLoS One. 2013 Sep 19;8(9):e75380. doi: 10.1371/journal.pone.0075380 (PMC3777905; doi:10.1371/journal.pone.0075380)

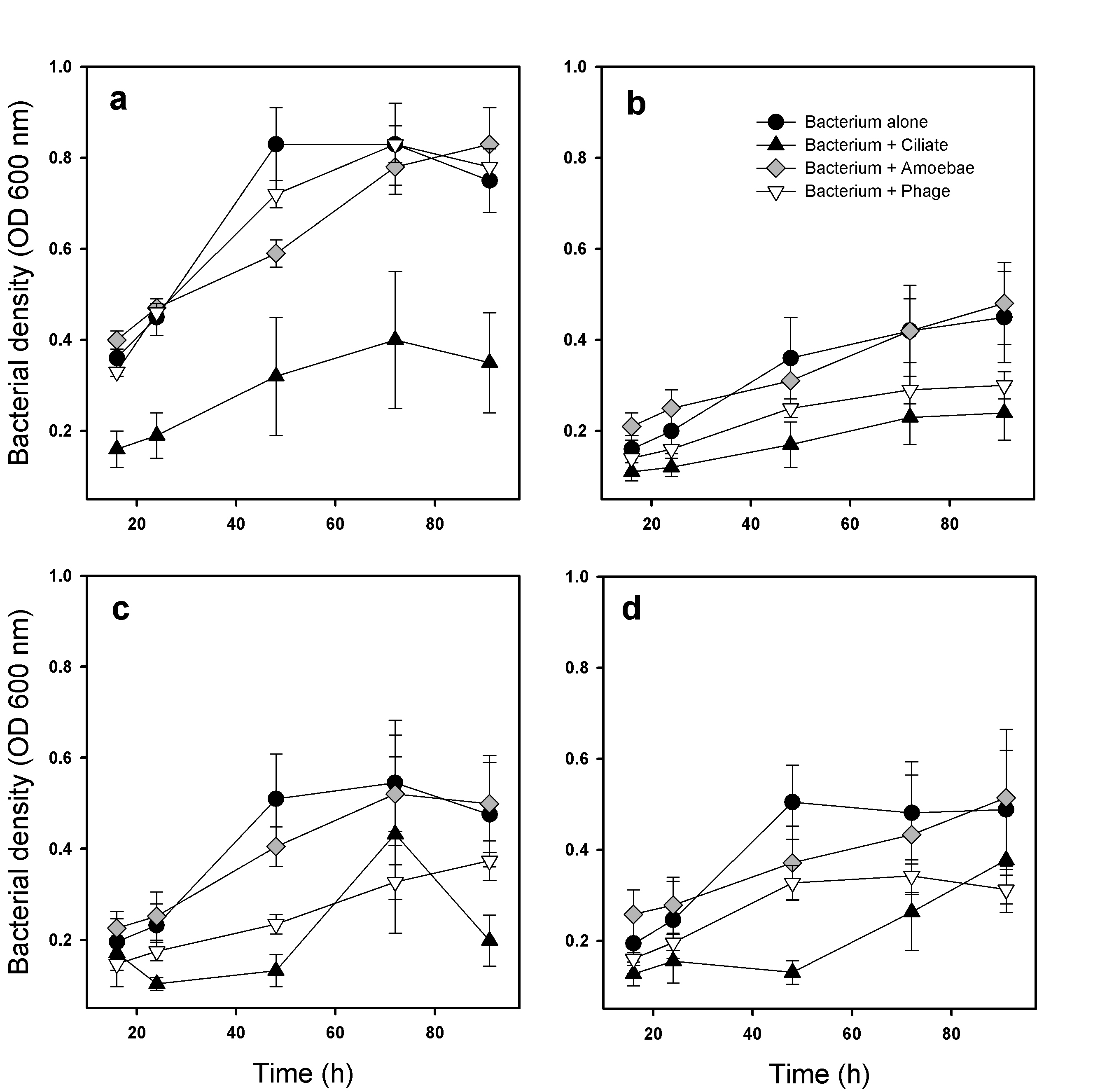

Supplement: Figure S1 — Bacterial growth in the absence and presence of different enemies. The growth of bacteria isolated from patients with intermittent colonisation (a) or chronic (b) CF-lung infection. Panels (c) and (d) show bacterial growth in the absence and presence of different enemies for yellow (c) and white (d) colony type means for chronic CF-patients. Error bars denote ± 1 SEM. (TIF) [file pone.0075380.s001.tif]
